# ViOTUcluster: A high‐speed, All‐in‐one pipeline for viromic analysis of metagenomic data

**DOI:** 10.1002/imo2.70023

**Published:** 2025-05-20

**Authors:** Sihang Liu, Yinyin Ye, Bing Guo, Yuxing Hu, Kaiyang Jiang, Chengyu Liang, Siqing Xia, Hong Wang

**Affiliations:** ^1^ State Key Laboratory of Water Pollution Control and Green Resource Recycling, College of Environmental Science and Engineering Tongji University Shanghai China; ^2^ Shanghai Institute of Pollution Control and Ecological Security Shanghai China; ^3^ Department of Civil, Structural and Environmental Engineering University at Buffalo Buffalo New York USA; ^4^ School of Sustainability, Civil and Environmental Engineering University of Surrey Guildford UK; ^5^ Jiaxing‐Tongji Environmental Research Institute Jiaxing Zhejiang Province China

## Abstract

ViOTUcluster is a user‐friendly, high‐speed, accurate, All‐in‐one solution that streamlines the entire viromic analysis workflow—from raw reads to the generation of viral operational taxonomic units tables, as well as other key viromic analysis tasks.

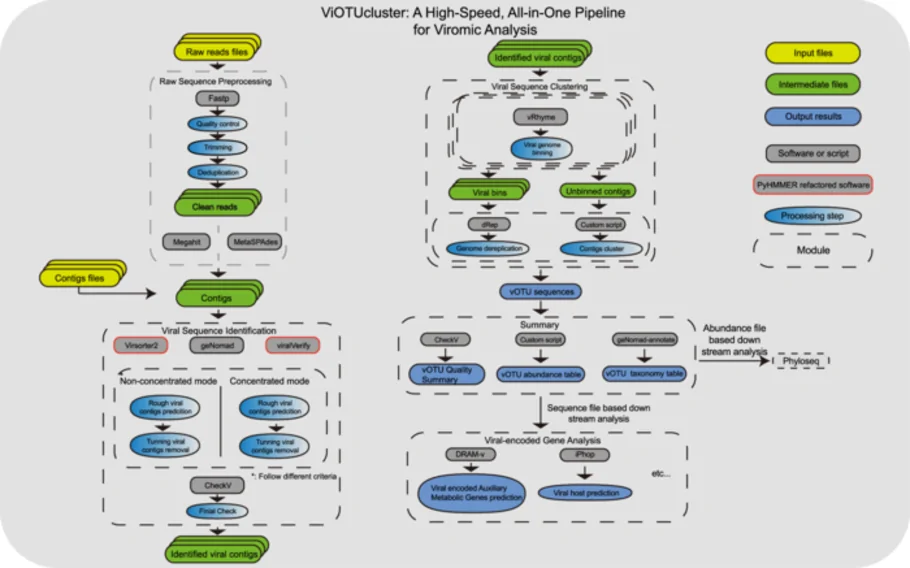

To the editor,

Recent advances in metagenomic sequencing technologies have further enabled the comprehensive exploration of viral communities across diverse environments, uncovering their vast genetic diversity and critical ecological functions. [1, 2, 3] Despite continued advances in novel bioinformatics tools for viral ecology research, the complexity of viromic data analysis workflows remains a significant challenge. For instance, the absence of standardized input and output formats across various software tools, combined with considerable variability in computational efficiency of software, creates substantial obstacles for researchers with limited bioinformatics expertise and/or access to computational resources.

Here, we introduce ViOTUcluster, a high‐speed and All‐in‐one solution for streamlined viromic analysis workflow. ViOTUcluster integrates the state‐of‐the‐art bioinformatic tools for viromic analysis, enabling the generation of viral operational taxonomic unit (vOTU) tables and the performance of genome‐level gene function analysis in a user‐friendly way. Through optimization of tool parameters and customization of viral identification criteria to accommodate samples with varying viral sequence abundances, ViOTUcluster demonstrates high analytical accuracy, achieving F1‐scores exceeding 0.83 in low‐viral abundance samples and 0.94 in high‐viral abundance samples. ViOTUcluster also enhances computational efficiency, reducing viral sequence identification time by up to 16.4‐fold through the integration of refactored VirSorter2 and viralVerify. In summary, ViOTUcluster offers an efficient, accurate, and standardized viromic analysis workflow that enables researchers to effectively analyze and interpret large‐scale viral sequencing data. The source code is available on GitHub (https://github.com/liusihang/ViOTUcluster), with detailed usage instructions on the project website.

## 1 RESULTS AND DISCUSSION

### Overview of ViOTUcluster workflow

ViOTUcluster comprises five modules for comprehensive viral analysis: raw sequence preprocessing, viral sequence identification, viral sequence clustering, summary generation, and analysis of viral‐encoded genes (Method detailed in Text S1). First, the **Raw Sequence Preprocessing** module performs quality assessment of raw sequencing data, followed by trimming to generate high‐quality reads for assembly. [4, 5, 6] In this module, users can choose either Megahit or metaSPAdes to assemble high‐quality contigs for samples. Previous studies have shown that Megahit and metaSPAdes outperform other assembly software, with metaSPAdes providing the highest overall effectiveness. [7] However, Megahit offers faster assembly with lower RAM usage. Users can choose either tool based on their preference. Second, the **Viral Sequence Identification** module has “Non‐concentrated” and “Concentrated” modes, designed for non‐concentrated samples and viral‐particle‐enriched samples, respectively. [2, 6, 8, 9] Providing these two modes enables analysis based on different viral contig abundance in different sample types, leading to improved precision and recall rates. Third, potential viral sequences from each sample are subsequently processed in the **Viral Sequence Clustering** module to generate viral bins and unbinned sequences. [10] Then, the bins and unbinned sequences from all samples are pooled and dereplicated according to the Uncultivated Virus Genome (MIUViG) standards, using dRep and a custom Python script to eliminate redundancy. [8, 11, 12] The resulting nonredundant vOTU file is subsequently used in the **Summary** module to generate a vOTU abundance table and assign taxonomy, and further to predict viral‐encoded AMGs and determine viral hosts in the **Viral‐encoded Gene Analysis** module. [1, 13] All output files are formatted for user convenience and are fully compatible with R packages such as phyloseq, facilitating seamless downstream viral ecological analysis.

### Performance metrics of viral‐sequence‐identification module

To evaluate the performance of the viral identification module in **ViOTUcluster**, we generated mock contig test sets from genomes in the NCBI RefSeq database (Text S1). Considering the widespread application of both viral‐like particle‐enriched samples (targeting the free viral particle fraction) and non‐concentrated samples (targeting the cellular fraction) in viromic studies, we developed two distinct analytical modes tailored specifically for these sample types. Each mode comprises two main steps: (1) viral sequence recovery and (2) nonviral sequence removal (Figure S1). Our results demonstrated that these two steps effectively enhanced the performance of the “Non‐concentrated” mode in low‐virus mock samples. Specifically, applying more stringent criteria during the viral sequence recovery step enabled accurate identification of an average of 90.7% of viral sequences, while misclassifying fewer than 3.3% of total nonviral sequences (Figure S2). Furthermore, the nonviral sequence removal step effectively reduced contamination, lowering the false positive rate by 15.5%–18.2%, with only 0.61%–0.76% of true viral sequences mistakenly removed (Figure S3). Collectively, compared to the “Concentrated” mode, the “Non‐concentrated” mode achieved superior performance in low‐virus samples by significantly reducing nonviral contamination, resulting in a 23.4%–25.4% decrease in false positive sequences, with less than a 1% reduction in recall rate (Figure S4). These results demonstrate the effectiveness of this analytical mode for accurately identifying viral sequences in samples with varying viral abundances.

**FIGURE 1:**
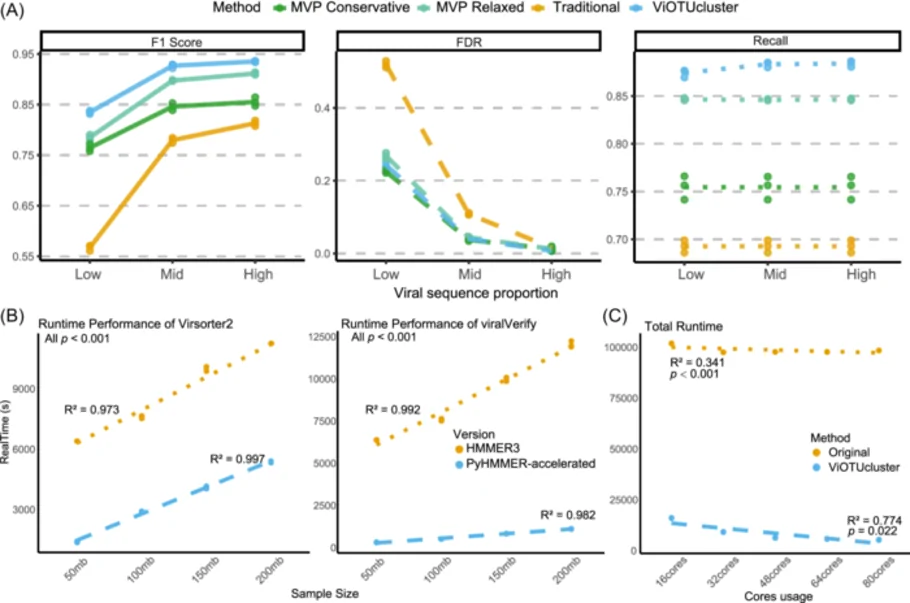
Evaluation of accuracy and runtime performance of ViOTUcluster. (A) Performance comparison between the traditional method, the Viral Sequence Identification module of ViOTUcluster, and the relaxed and conservative modes of MVP using mock viral sequence samples. Three viral content levels were evaluated: Low (10% viral sequences), Mid (50%), and High (90%). For each level, performance metrics were averaged over three replicates, with error bars representing the standard deviation. Evaluation metrics include F1 score, false discovery rate (FDR), and recall. (B) Runtime performance comparison between the original and refactored versions of VirSorter2 and viralVerify using mock samples of varying sizes. The mock datasets were constructed by randomly selecting viral sequences from the NCBI Virus database. A sample size of 50 Mb corresponds to approximately 50 million base pairs, with larger sizes scaled proportionally. (C) Total runtime comparison between the ViOTUcluster viral sequence prediction module and the original method across different numbers of CPU cores (16–80). In the original workflow, geNomad, VirSorter2, and viralVerify were executed sequentially on each sample, while ViOTUcluster processed the same samples in parallel across the three tools. All tests were conducted on 10 mock samples, each containing 4000 viral contigs. The coefficient of determination (*R*²) and *p*‐value shown in panels (B) and (C) were derived from a linear regression model, with the *p*‐value representing the statistical significance of the regression slope based on a *t*‐test.

To comprehensively evaluate the overall performance of the **Viral Sequence Identification** module, the viral identification results obtained by ViOTUcluster were compared with those from the “traditional method,” a combination of three widely used tools (VirSorter2, VIBRANT, and DeepVirFinder; detailed methods provided in Text S1) and a recently published viral identification pipeline, “MVP”, which also supports cross‐sample analysis of viral community datasets. [14] To thoroughly assess the true capabilities of “MVP,” tests were run under both its Relaxed and Conservative modes. Using the F1 score as a comprehensive metric for viral identification performance, our analysis revealed that ViOTUcluster achieved the highest performance among all evaluated methods in low‐viral‐abundance samples, with MVP in its Relaxed mode ranking second (Figure 1A). Both ViOTUcluster and MVP demonstrated improved identification performance compared to the “traditional method.” Notably, ViOTUcluster consistently achieved higher recall rates than both MVP modes, with improvements ranging from 3.4% to 14.4%. Although the Conservative mode of MVP achieved the lowest FDR, this came at the expense of a relatively low recall rate. For mid‐ and high‐viral‐abundance samples, ViOTUcluster and MVP Relaxed mode performed comparably, achieving F1 scores of 0.91–0.94 and 0.89–0.91, respectively.

Collectively, these results show that ViOTUcluster achieved F1 scores above 0.83 in mock low‐virus samples and over 0.94 in mock high‐virus samples, demonstrating higher average performance across all mock contig samples with varying viral abundances (Figure 1A). Compared to the “traditional method” and “MVP,” ViOTUcluster improved F1 scores by 17.28%–50.91% and 2.4%–9.7%, respectively. Given the overlap in viral identification software among ViOTUcluster, MVP, and traditional methods, these findings highlight the benefits of integrating multiple viral prediction tools and optimizing parameters to enhance both the accuracy and yield of identified viral sequences.

### ViOTUcluster with refactored tools reduces computational time for large‐scale analyses

Due to inherent I/O constraints in its search algorithm, viral prediction using HMMER remains a time‐consuming process, particularly in the context of the continuously expanding viral reference databases. For instance, Virsorter2 demonstrated that viral protein annotation using larger viral Hidden Markov Model (HMM) databases was the most time‐consuming step, consuming over 90% CPU time during the entire process. [9] PyHMMER is a recently developed Python package based on HMMER, designed to enhance computational efficiency through an optimized new threading strategy, while producing results identical to the original HMMER tool. [15] Here, we refactored VirSorter2 and viralVerify by replacing hmmsearch in VirSorter2 and viralVerify with PyHMMER search. By pre‐loading target sequence files into memory, the performance issues caused by I/O bound limitations in the original version of HMMER are resolved. In addition, the HMM database was recompiled to binary format to reduce load time, which further enhanced computational efficiency. Compared to the original versions, the refactored VirSorter2 and viralVerify demonstrated substantial time reductions in classifying single contig files of varying sizes (approximate 50–200 million bp), with average speed‐ups from 2 to 4.5 times for VirSorter2 and from 9.8 to 16.1 times for viralVerify (Figure 1B). Overall, ViOTUcluster demonstrated an efficiency improvement of 3.4 to 5.5 times in handling contig files of different sample sizes (Figure S5).

The **Viral Sequence Identification** module in ViOTUcluster can parallelize sample processing processes (i.e., VirSorter2, viralVerify, and geNomad) and automatically allocates threads across tasks to further reduce the runtime of single‐threaded operations. To evaluate multithreading efficiency, 16, 32, 48, 64, and 80 threads were tested to run the **Viral Sequence Identification** module on ten contig files, each containing 4000 randomly selected sequences from the NCBI RefSeq database. Benchmark results indicated that the **Viral Sequence Identification** module in ViOTUcluster demonstrated higher multithreading efficiency compared to the sequential analysis performed by VirSorter2, viralVerify, and geNomad in the non‐refactored version, with runtime improvements ranging from 6.1 to 16.4 times depending on the number of threads used (Figure 1C). To further evaluate the scalability and robustness of ViOTUcluster, its performance was tested across various Linux systems and hardware configurations, which shown consistent improvements as described above (Table S1 and Figure S6). These results suggest that ViOTUcluster can be efficiently utilized on hardware with different thread configurations and handle larger datasets effectively.

### Benchmarking with real‐world metagenomic data

To evaluate ViOTUcluster's performance on real metagenomic datasets from natural and engineered environments, four ocean samples (70–90 million reads each) and four wastewater treatment plant (WWTP) samples (113–135 million reads each) were analyzed using both ViOTUcluster and MVP. MEGAHIT assembled 676,194 to 903,527 contigs from the WWTP samples and 610,891 to 958,480 contigs from the ocean samples.

**FIGURE 2:**
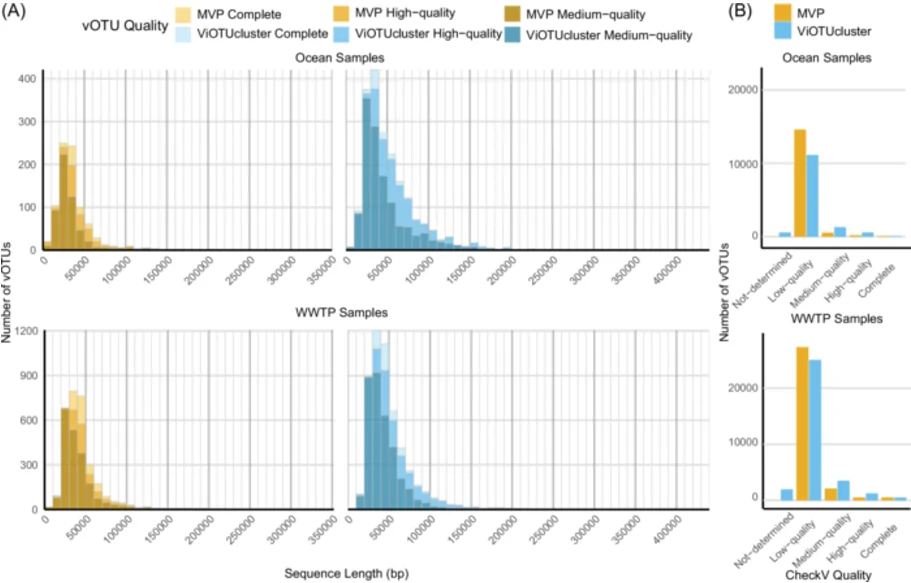
Reconstructed viral operational taxonomic units (vOTUs) quality in ocean and WWTP samples. (A) Sequence length distribution of vOTUs reconstructed by MVP and ViOTUcluster from ocean and WWTP samples. Bars represent the number of vOTUs binned by length and stratified by CheckV‐assigned quality levels. (B) Overall distribution of vOTUs across CheckV quality categories, grouped by data set and sample type.

When applied in concentrated mode, ViOTUcluster recovered 13,704 “species‐level” vOTUs from ocean samples and 32,281 from WWTP samples, following the steps of binning, genome clustering, and filtering out contigs shorter than 5 kbp. MVP pipeline identified 15,467 vOTUs from ocean samples and 30,536 from WWTP samples, using relaxed‐mode viral prediction and conservative filtering as recommended by its authors (Figure S7). The quality of the recovered vOTUs was further evaluated using CheckV. ViOTUcluster reconstructed a significantly higher number of medium‐, high‐, and complete‐quality vOTUs, which also exhibited longer contig lengths compared to vOTUs obtained from MVP (Figure 2A and Figure S8). Notably, ViOTUcluster recovered a substantial number of high‐quality and complete viral genomes exceeding 50 kb. Meanwhile, ViOTUcluster reconstructed fewer low‐quality vOTUs, possibly because its binning function leverages low‐quality contigs to enhance genome reconstruction (Figure 2B). According to geNomad annotations, *Caudoviricetes* was the dominant viral class across all samples for both pipelines. Notably, even with the integration of multiple viral identification tools, ViOTUcluster used substantially shorter runtimes than the MVP pipeline (66 h 12 min for ViOTUcluster and 137 h 19 min for MVP) when tested on a system with 104 CPUs, which is attributed to its optimized resource efficiency. These results highlight ViOTUcluster's ability to efficiently process diverse environmental metagenomic datasets, yielding high‐quality vOTUs and longer contig lengths with substantially reduced computational time.

### Current limitations and future developments

ViOTUcluster has demonstrated its reliability in identifying viral sequences in both simulated and real‐world datasets, achieving optimized computational runtime through effective resource utilization. However, certain limitations merit consideration. First, the NCBI RefSeq database currently contains a relatively small proportion of uncultured viral genomes, meaning test datasets are largely dominated by reference genomes from isolated viruses. As a result, the identification performance on mock contigs may not fully reflect ViOTUcluster's capacity to detect uncultured viruses. As the database grows to encompass a greater number of uncultured viral genomes, ViOTUcluster's predictive accuracy can be more thoroughly and robustly validated. Additionally, ViOTUcluster is presently restricted to Linux‐based systems, potentially limiting accessibility for researchers using other operating systems. Future development efforts will enhance cross‐platform compatibility via Docker to support wider adoption.

## 2 CONCLUSION

Advances in metagenomic sequencing and bioinformatics have significantly expanded our ability to explore uncultured viral communities. However, the rapid expansion of viromics poses significant challenges for researchers, especially those with limited bioinformatics expertise, in organizing data and integrating outputs from diverse analytical tools. To address these challenges, we present ViOTUcluster—an innovative, user‐friendly, and modular pipeline for viromic analysis—designed to perform major tasks in viral metagenomic data processing starting from raw sequencing reads. By providing a comprehensive, accurate, and reproducible workflow, ViOTUcluster facilitates virome analysis across a wide range of environmental contexts. Moving forward, ViOTUcluster will continue to evolve by integrating emerging tools and methodologies, ensuring it remains at the forefront of viromics research. This commitment ensures that it will remain a pivotal resource for the research community, enabling more precise and comprehensive investigations into viral ecology.

## AUTHOR CONTRIBUTIONS

**Sihang Liu**: Conceptualization; investigation; methodology; software; data curation; formal analysis; validation; visualization; writing—original draft; writing—review and editing; project administration; resources. **Yinyin Ye**: Writing—review and editing. **Bing Guo**: Writing—review and editing. **Yuxing Hu**: Writing—review and editing. **Kaiyang Jiang**: Writing—review and editing. **Chengyu Liang**: Writing—review and editing. **Siqing Xia**: Funding acquisition; supervision. **Hong Wang**: Writing—review and editing; funding acquisition; project administration; supervision.

## CONFLICT OF INTEREST STATEMENT

The authors declare no conflicts of interest.

## ETHICS STATEMENT

No animals or humans were involved in this study.

## Supporting information


**Text S1:** Details of Methods and Material.
**Figure S1:** Workflow of viral sequence identification module.
**Figure S2:** The number of contigs classified as viruses in the first step of ViOTUcluster viral identification module.
**Figure S3:** Effect of additional removal rulesets on the removal of non‐viral sequences across samples with varying viral ratios.
**Figure S4:** Performance metric of ViOTUcluster “Concentrate” mode and “Non‐concentrated” mode for mock samples with low‐virus abundance.
**Figure S5:** Runtime performance comparison of the viral sequence identification module using the original and refactored versions of VirSorter2 and viralVerify under mock samples of varying sizes.
**Figure S6:** Total runtime comparison between the ViOTUcluster viral sequence prediction module and the original method across different numbers of CPU cores under four hardware configurations.
**Figure S7:** The number of identified vOTUs in each sample between MVP and ViOTUcluster.
**Figure S8:** Violin plot comparing medium‐, high‐, and complete vOTU sequence lengths obtained using MVP and ViOTUcluster in ocean and WWTP samples.
**Figure S9:** The number of contigs classified as plasmids by geNomad and viralVerify in mock samples with different viral ratios.
**Figure S10:** Host distribution of NCBI virus Refseq database.

**Table S1:** Hardware Configuration Employed for ViOTUcluster Testing.
**Table S2:** Core tools used in ViOTUcluster.
**Table S3:** Detailed key parameters of viral identification tools.
**Table S4:** Summary of the usage of viral sequence identification software.

## Data Availability

The data that support the findings of this study are openly available in the Raw data for ViOTUcluster at https://zenodo.org/records/15036758. The data and scripts utilized in ViOTUcluster are publicly available on GitHub (https://github.com/liusihang/ViOTUcluster), along with comprehensive tutorials and example workflows. The data that support the findings of this study, example data, and corresponding results used in the manuscript are openly accessible via Zenodo (https://zenodo.org/records/15036758). Supplementary materials (text, figures, tables, graphical abstract, slides, videos, Chinese translated version, and update materials) may be found in the online DOI or iMetaOmics http://www.imeta.science/imetaomics/.
