# Supplementary material for: ViOTUcluster: A high‐speed, All‐in‐one pipeline for viromic analysis of metagenomic data
Source: IMetaOmics. 2025 May 20;2(2):e70023. doi: 10.1002/imo2.70023 (PMC12806464; doi:10.1002/imo2.70023)
Supplement: Supplementary file 1 — Text S1: Details of Methods and Material. Figure S1: Workflow of viral sequence identification module. Figure S2: The number of contigs classified as viruses in the first step of ViOTUcluster viral identification module. Figure S3: Effect of additional removal rulesets on the removal of non‐viral sequences across samples with varying viral ratios. Figure S4: Performance metric of ViOTUcluster “Concentrate” mode and “Non‐concentrated” mode for mock samples with low‐virus abundance. Figure S5: Runtime performance comparison of the viral sequence identification module using the original and refactored versions of VirSorter2 and viralVerify under mock samples of varying sizes. Figure S6: Total runtime comparison between the ViOTUcluster viral sequence prediction module and the original method across different numbers of CPU cores under four hardware configurations. Figure S7: The number of identified vOTUs in each sample between MVP and ViOTUcluster. Figure S8: Violin plot comparing medium‐, high‐, and complete vOTU sequence lengths obtained using MVP and ViOTUcluster in ocean and WWTP samples. Figure S9: The number of contigs classified as plasmids by geNomad and viralVerify in mock samples with different viral ratios. Figure S10: Host distribution of NCBI virus Refseq database. [file IMO2-2-e70023-s002.docx]

**Supporting Information to ViOTUcluster: A High-Speed, All-in-One Pipeline for Viromic Analysis of Metagenomic Data**

**Running title:** ViOTUcluster: All-in-One Pipeline for Viromic Analysis of Metagenomic Data

Sihang Liu^1,2^, Yinyin Ye^3^, Bing Guo^4^, Yuxing Hu^1,2^, Kaiyang Jiang^1,2^, Chengyu Liang^1,2^, Siqing Xia^1,2*^, Hong Wang^1,2,5*^

^1^State Key Laboratory of Water Pollution Control and Green Resource Recycling, College of Environmental Science and Engineering, Tongji University, Shanghai 200092, China

^2^Shanghai Institute of Pollution Control and Ecological Security, Shanghai 200092, China

^3^Department of Civil, Structural and Environmental Engineering, University at Buffalo, Buffalo, New York 14260, United States

^4^School of Sustainability, Civil and Environmental Engineering, University of Surrey, Guildford GU2 7XH, UK;

^5^Jiaxing-Tongji Environmental Research Institute, 1994 Linggongtang Road, Jiaxing 314051, Zhejiang Province, China

*Correspondence: [siqingxia@tongji.edu.cn](mailto:hongwang@tongji.edu.cn) (Siqing Xia), [hongwang@tongji.edu.cn](mailto:hongwang@tongji.edu.cn) (Hong Wang)

**SUMMARY: This 17-page supporting information includes:**

Detailed materials and methods: Details of Methods and Material (Text S1); Workflow of viral sequence identification module (Figure S1); The number of contigs classified as viruses in the first step of ViOTUcluster viral identification module (Figure S2); Effect of additional removal rulesets on the removal of non-viral sequences across samples with varying viral ratios (Figure S3); Performance metric of ViOTUcluster “Concentrate” mode and “Non-concentrated” mode for mock samples with low-virus abundance (Figure S4); Runtime performance comparison of the viral sequence identification module using the original and refactored versions of VirSorter2 and viralVerify under mock samples of varying sizes (Figure S5); Total runtime comparison between the ViOTUcluster viral sequence prediction module and the original method across different numbers of CPU cores under four hardware configurations (Figure S6); The number of identified vOTUs in each sample between MVP and ViOTUcluster (Figure S7); Violin plot comparing medium-, high-, and complete vOTU sequence lengths obtained using MVP and ViOTUcluster in ocean and WWTP samples (Figure S8); The number of contigs classified as plasmids by geNomad and viralVerify in mock samples with different viral ratios (Figure S9); Host distribution of NCBI virus Refseq database (Figure S10).

**Text S1. Supplementary methods**

**Overview of the pipeline**

ViOTUcluster is a streamlined and user-friendly pipeline designed to integrate multiple state-of-the-art viromic analysis tools, enabling fast and automated analysis from both DNA and RNA metagenomic data. The pipeline comprises five distinct modules, with the core tools for each detailed in the Supporting Information (SI, Table S2). Detailed installation instructions for ViOTUcluster are available on GitHub (https://github.com/liusihang/ViOTUcluster), and the software can be easily installed via the setup script. All benchmarking analyses were conducted using ViOTUcluster v0.5.2.1.

**Raw sequence processing and assembly**

The quality of raw reads is evaluated using fastp (v0.23.4) with default settings. This process includes automatic adapter removal, quality trimming, read filtering, and base correction, ensuring the generation of high-quality reads. [1] Contig assembly can be performed independently for each sample, using either metaSPAdes (v4.0.0) or MEGAHIT (v1.2.9), based on user preference. [2,3] Finally, contigs exceeding a user-defined length threshold are selected for viral sequence identification.

**Viral sequence identification**

To further expand the viral recovery rate and harness the complementary strengths of diverse viral identification algorithms, we selected three state-of-the-art tools with distinct algorithms: VirSorter2 (v2.2.4), geNomad (v1.7.6), and viralVerify (v1.1). [4−6] Virsorter2 employs a set of multi-classifier Random Forest models to classify different viruses, geNomad combines an alignment-free classifier and a gene-based classifier to improve classification performance, and viralVerify utilizes a Naive Bayes classifier to identify viral contigs. Each of these tools has demonstrated reliable performance on mock viral samples, achieving an F1 score above 0.85 in previous studies. [4−9] Additionally, CheckV (v1.0.3) was used to assess viral genome quality. [10]

Viral sequence identification involves two primary steps: (1) viral sequence recovery and (2) non-viral sequence removal (Figure S1). During the viral sequence recovery step, each tool applies specific criteria tailored to non-concentrated and concentrated metagenomic samples, referred to as “Rulesets”. Detailed parameters and rules for “Non-concentrated” and “Concentrated” modes are provided in Table S3 and Figure S1, respectively. In brief, three viral identification tools were applied in the “Concentrated” mode for viral-particle enriched samples, using looser criteria to recover a more comprehensive set of viral sequences. In the “Non-concentrated” mode, the same tools were applied with stricter criteria to minimize contamination from the high ratio of non-viral sequences. Notably, through utilize the plasmid identification by geNomad and viralVerify, non-viral contamination be further reduced (Figure S9).

To compare performance of viral prediction module of ViOTUcluster, three widely used tools over the past two years (i.e., VirSorter2, VIBRANT, and DeepVirFinder) were employed as controls in this study, collectively referred to as the “Traditional method” (Table S4). [4,11,12] The “traditional method” ruleset was applied to contigs prefiltered at ≤5 kb, using filter criteria from previous studies when viral contig quality information was available in the tool outputs: (i) a VirSorter2 score > 0.9 and *P* < 0.05; (ii) classification as a virus by VIBRANT; and (iii) a DeepVirFinder score > 0.9 and *P* < 0.05. [11,13−17]

To achieve more precise genome-level clustering, vRhyme (v1.1.0) was used to group sequences from the same genome into viral metagenome-assembled genome (vMAG). [18] Contigs shorter than 5 kb and those not assigned to any bin were removed.

**Viral sequence clustering and taxonomy classification**

Custom Python scripts and dRep (v3.4.2) were used to cluster unbinned viral sequences and viral bins based on an average nucleotide identity (ANI) of 95% across at least 85% of the sequence length. [10,19] All non-redundant viral bins and unbinned viral sequences were consolidated as vOTUs for downstream analysis. Taxonomy of vOTUs were assigned by geNomad based on the taxonomic lineages defined in the Virus Metadata Repository of the International Committee on Taxonomy of Viruses (ICTV’s VMR number 19). [20]

**Virus-carried gene identification and viral host prediction**

In case users wish to identify potential Auxiliary Metabolic Genes (AMGs) and predict viral hosts, the Distilled and Refined Annotation of Metabolism (DRAM-v) tool was used to predict AMGs carried by vOTUs, while iPHoP (v1.3.3) was employed for viral host prediction as an optional step. [21,22]

**Viral abundance estimation**

Custom scripts were developed to estimate viral sequence abundance in Transcripts Per Million (TPM). Briefly, clean reads from each sample were mapped to sequences in the final vOTU file using BWA-MEM (v0.7.17). [21] Sequence coverage for each vOTU was calculated using CoverM (v1.2.0). [22] Based on the coverage data of each sample, a custom Python script was used to calculate the TPM of vOTUs and summarize the results into a total abundance file.

**Benchmarking for ViOTUcluster**

To evaluate the performance of viral identification rulesets, mock contig testing sets were generated, comprising viral, plasmid, bacterial, archaeal, fungal, and protist reference genomes. To simulate varying viral read ratios in metagenomes, three sample types were generated: low-virus, mid-virus, and high-virus, corresponding to virus-to-non-virus sequence ratios of 1:10, 1:1, and 10:1, respectively. Each sample contained 2,000 viral sequences randomly sampled from the NCBI virus reference database (<https://www.ncbi.nlm.nih.gov/labs/virus>, accessed April 2024). Host distribution of virus in NCBI virus Refseq database was shown in Figure S10. Non-viral sequences were randomly selected from the NCBI reference sequence database (RefSeq, Release 225) using a custom Python script. The non-viral component of the testing sets was designed to include approximately 70% bacterial, 15% archaeal, 6% plasmid, 6% fungal, and 3% protist sequences. The total sequence counts in the three tests were 2,200, 4,000, and 22,000, respectively. For each condition, triplicate samples were generated to ensure reproducibility and strengthen the robustness of the results across replicates. Notably, the viral sequences were identical across the three sample types, ensuring direct comparison of viral outcomes across different ratios.

To further evaluate the performance of ViOTUcluster against other viral identification pipelines, we analyzed real metagenomic datasets from marine environments and wastewater treatment plants, representing natural and engineered systems, respectively. Unless otherwise specified, all analyses were conducted using default parameters on a Dell T7920 server equipped with dual Intel Xeon Gold 6268L processors and 256 GB RAM, running Ubuntu 22.04. Detail test results were provided in Zenodo (https://zenodo.org/records/15036758).

**REFERENCES**

1. Shifu Chen. 2023. “Ultrafast one-pass FASTQ data preprocessing, quality control, and deduplication using fastp.” *iMeta* 2: e107. https://doi.org/10.1002/imt2.107

2. Andrey Prjibelski, Dmitry Antipov, Dmitry Meleshko, Alla Lapidus, and Anton Korobeynikov. 2020. “Using SPAdes De Novo Assembler.” *Current Protocols in Bioinformatics* 70: e102. https://doi.org/10.1002/cpbi.102

3. Dinghua Li, Chi-Man Liu, Ruibang Luo, Kunihiko Sadakane, and Tak-Wah Lam. 2015. “MEGAHIT: an ultra-fast single-node solution for large and complex metagenomics assembly via succinct *de Bruijn* graph.” *Bioinformatics* 31: 1674–1676. https://doi.org/10.1093/bioinformatics/btv033

4. Jiarong Guo, Ben Bolduc, Ahmed A. Zayed, Arvind Varsani, Guillermo Dominguez-Huerta, Tom O. Delmont, Akbar Adjie Pratama, M. Consuelo Gazitúa, Dean Vik, Matthew B. Sullivan, and Simon Roux. 2021. “VirSorter2: a multi-classifier, expert-guided approach to detect diverse DNA and RNA viruses.” *Microbiome* 9: 37. https://doi.org/10.1186/s40168-020-00990-y

5. Antonio Pedro Camargo, Simon Roux, Frederik Schulz, Michal Babinski, Yan Xu, Bin Hu, Patrick S. G. Chain, Stephen Nayfach, and Nikos C. Kyrpides. 2023. “Identification of mobile genetic elements with geNomad.” *Nature Biotechnology* 1–10. https://doi.org/10.1038/s41587-023-01953-y

6. Dmitry Antipov, Mikhail Raiko, Alla Lapidus, and Pavel A. Pevzner. 2020. “Metaviral SPAdes: assembly of viruses from metagenomic data.” *Bioinformatics* 36: 4126–4129. https://doi.org/10.1093/bioinformatics/btaa490

7. Ling-Yi Wu, Yasas Wijesekara, Gonçalo J. Piedade, Nikolaos Pappas, Corina P. D. Brussaard, and Bas E. Dutilh. 2024. “Benchmarking bioinformatic virus identification tools using real-world metagenomic data across biomes.” *Genome Biology* 25: 97. https://doi.org/10.1186/s13059-024-03236-4

8. Bridget Hegarty, James Riddell V, Eric Bastien, Kathryn Langenfeld, Morgan Lindback, Jaspreet S. Saini, Anthony Wing, Jessica Zhang, and Melissa Duhaime. 2024. “Benchmarking informatics approaches for virus discovery: caution is needed when combining in silico identification methods.” *mSystems* 9: e01105-23. https://doi.org/10.1128/msystems.01105-23

9. Joachim Johansen, Damian R. Plichta, Jakob Nybo Nissen, Marie Louise Jespersen, Shiraz A. Shah, Ling Deng, Jakob Stokholm, Hans Bisgaard, Dennis Sandris Nielsen, Søren J. Sørensen, and Simon Rasmussen. 2022. “Genome binning of viral entities from bulk metagenomics data.” *Nature Communications* 13: 965. https://doi.org/10.1038/s41467-022-28581-5

10. Stephen Nayfach, Antonio Pedro Camargo, Frederik Schulz, Emiley Eloe-Fadrosh, Simon Roux, and Nikos C. Kyrpides. 2021. “CheckV assesses the quality and completeness of metagenome-assembled viral genomes.” *Nature Biotechnology* 39: 578–585. https://doi.org/10.1038/s41587-020-00774-7

11. Kristopher Kieft, Zhichao Zhou, and Karthik Anantharaman. 2020. “VIBRANT: automated recovery, annotation and curation of microbial viruses, and evaluation of viral community function from genomic sequences.” *Microbiome* 8: 90. https://doi.org/10.1186/s40168-020-00867-0

12. Jie Ren, Kai Song, Chao Deng, Nathan A. Ahlgren, Jed A. Fuhrman, Yi Li, Xiaohui Xie, Ryan Poplin, and Fengzhu Sun. 2020. “Identifying viruses from metagenomic data using deep learning.” *Quantitative Biology* 8: 64–77. https://doi.org/10.1007/s40484-019-0187-4

13. Aixi Tang, Jiayu Zhang, Jin Huang, Yu Deng, Dou Wang, Pingfeng Yu, Renxin Zhao, Yulin Wang, Zihan Chen, Tong Zhang, and Bing Li. 2024. “Decrypting the viral community in aerobic activated sludge reactors treating antibiotic production wastewater.” *Water Research* 122253. https://doi.org/10.1016/j.watres.2024.122253

14. Wei Liu, Chenyang Xu, Tianle Li, Zhengran Ren, Shan Hao, Zhan Chen, Xia Huang, and Xianghua Wen. 2024. “Temporal Dynamics and Contribution of Phage Community to the Prevalence of Antibiotic Resistance Genes in a Full-Scale Sludge Anaerobic Digestion Plant.” *Environmental Science & Technology* 58: 6296–6304. https://doi.org/10.1021/acs.est.4c00712

15. Jiayu Zhang, Aixi Tang, Tao Jin, Deshou Sun, Fangliang Guo, Huaxin Lei, Lin Lin, Wensheng Shu, Pingfeng Yu, Xiaoyan Li, and Bing Li. 2024. “A panoramic view of the virosphere in three wastewater treatment plants by integrating viral‐like particle‐concentrated and traditional non‐concentrated metagenomic approaches.” *iMeta* 3: e188. https://doi.org/10.1002/imt2.188

16. Tianyi Chen, Chunfang Deng, Zongzhi Wu, Tang Liu, Yuanyan Zhang, Xuming Xu, Xiaohui Zhao, Jiarui Li, Shaoyang Li, Nan Xu, and Ke Yu. 2023. “Metagenomic analysis unveils the underexplored roles of prokaryotic viruses in a full-scale landfill leachate treatment plant.” *Water Research* 245: 120611. https://doi.org/10.1016/j.watres.2023.120611

17. Wenqing Hong, Hong Mei, Xianyang Shi, Xiaoxing Lin, Shuijing Wang, Renjie Ni, Yan Wang, and Liyan Song. 2024. “Viral community distribution, assembly mechanism, and associated hosts in an industrial park wastewater treatment plant.” *Environmental Research* 247: 118156. https://doi.org/10.1016/j.envres.2024.118156

18. Kristopher Kieft, Alyssa Adams, Rauf Salamzade, Lindsay Kalan, and Karthik Anantharaman. 2022. “vRhyme enables binning of viral genomes from metagenomes.” *Nucleic Acids Research* 50: e83–e83. https://doi.org/10.1093/nar/gkac341

19. Matthew R. Olm, Christopher T. Brown, Brandon Brooks, and Jillian F. Banfield. 2017. “dRep: a tool for fast and accurate genomic comparisons that enables improved genome recovery from metagenomes through de-replication.” *The ISME Journal* 11: 2864–2868. https://doi.org/10.1038/ismej.2017.126

20. Jens H. Kuhn. 2021. “Virus Taxonomy.” *Encyclopedia of Virology* 28. https://doi.org/10.1016/B978-0-12-809633-8.21231-4

21. Heng Li. 2013. Aligning sequence reads, clone sequences and assembly contigs with BWA-MEM. *arXiv*. https://doi.org/10.48550/ARXIV.1303.3997

22. Donovan H. Parks, Michael Imelfort, Connor T. Skennerton, Philip Hugenholtz, and Gene W. Tyson. 2015. “CheckM: assessing the quality of microbial genomes recovered from isolates, single cells, and metagenomes.” *Genome Research* 25: 1043–1055. https://doi.org/10.1101/gr.186072.114

**Supplementary Figures**


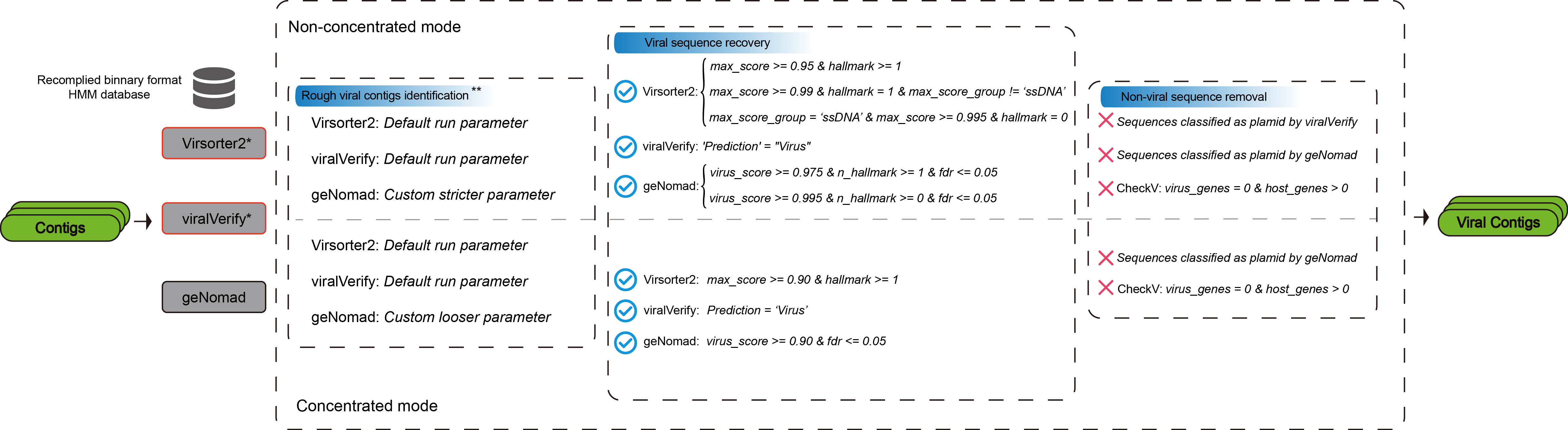


**Figure S1 Workflow of Viral Sequence identification module.** *: HMMER3 function in Virsorter2 and viralVerify was replaced by PyHMMER in our pipeline, and the HMM database was recompiled to binary format for faster loading. **: Detailed run parameters were provided in Table S3.


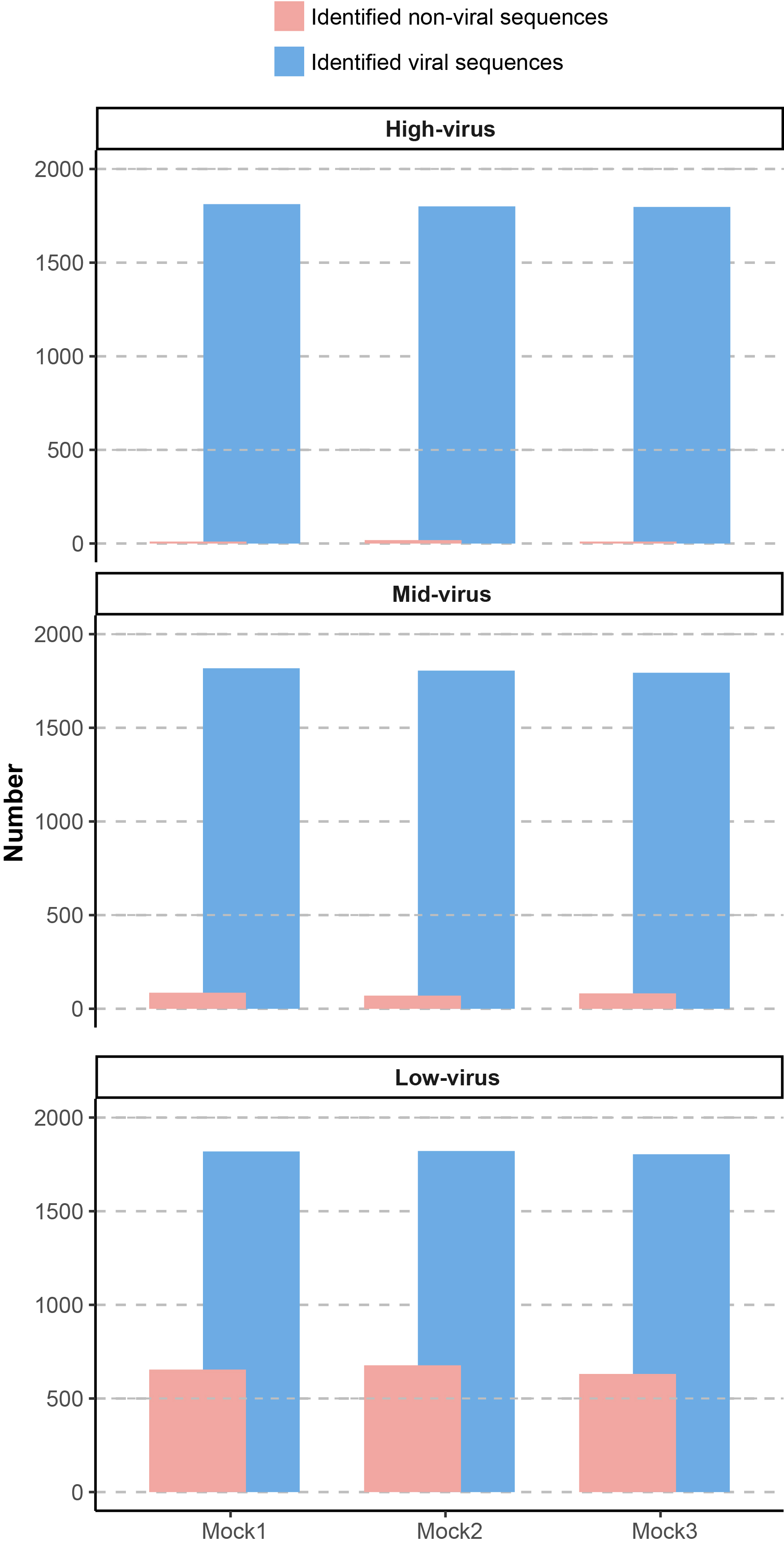


**Figure S2 The number of contigs classified as viruses in the first step of ViOTUcluster viral identification module.** The “Non-concentrated” mode was used for mock low-virus samples (Low: 2000 viral sequences + 20000 non-viral sequences), while the “Concentrated” mode was used for mock mid- (Mid: 2000 viral sequences + 2000 non-viral sequences) and high-virus samples (High: 2000 viral sequences + 200 non-viral sequences). Mock1, Mock2, and Mock3 represent the triplicates of samples with a specific viral ratio.


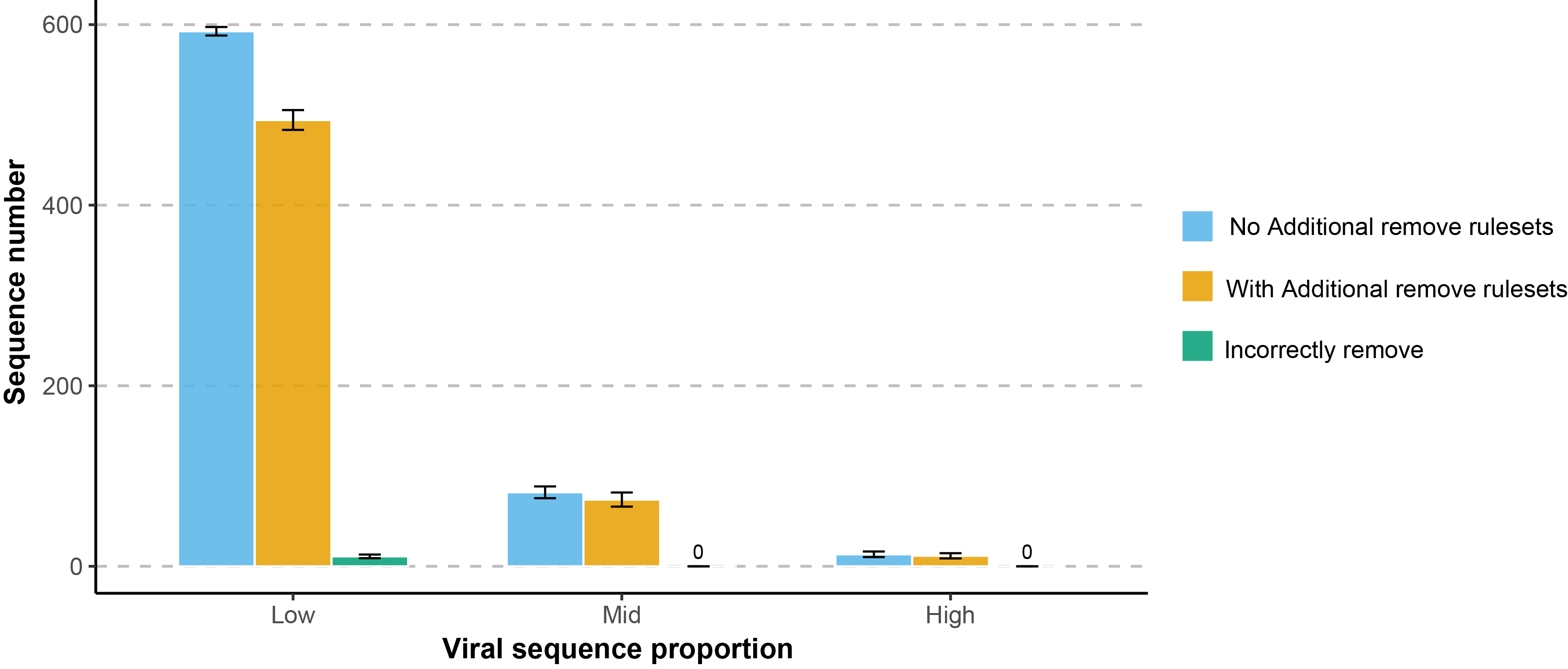


**Figure S3 Effect of additional removal rulesets on the removal of non-viral sequences across samples with varying viral ratios (Low: 2000 viral sequences + 20000 non-viral sequences, Mid: 2000 viral sequences + 2000 non-viral sequences, High: 2000 viral sequences + 200 non-viral sequences).** Bars represent the sequence counts without application of additional removal rulesets (No additional removal rulesets), with additional removal rulesets (With additional removal rulesets), and sequences incorrectly removed when additional removal rulesets are applied (Incorrectly removed). Error bars indicate the standard deviation across replicates.

**
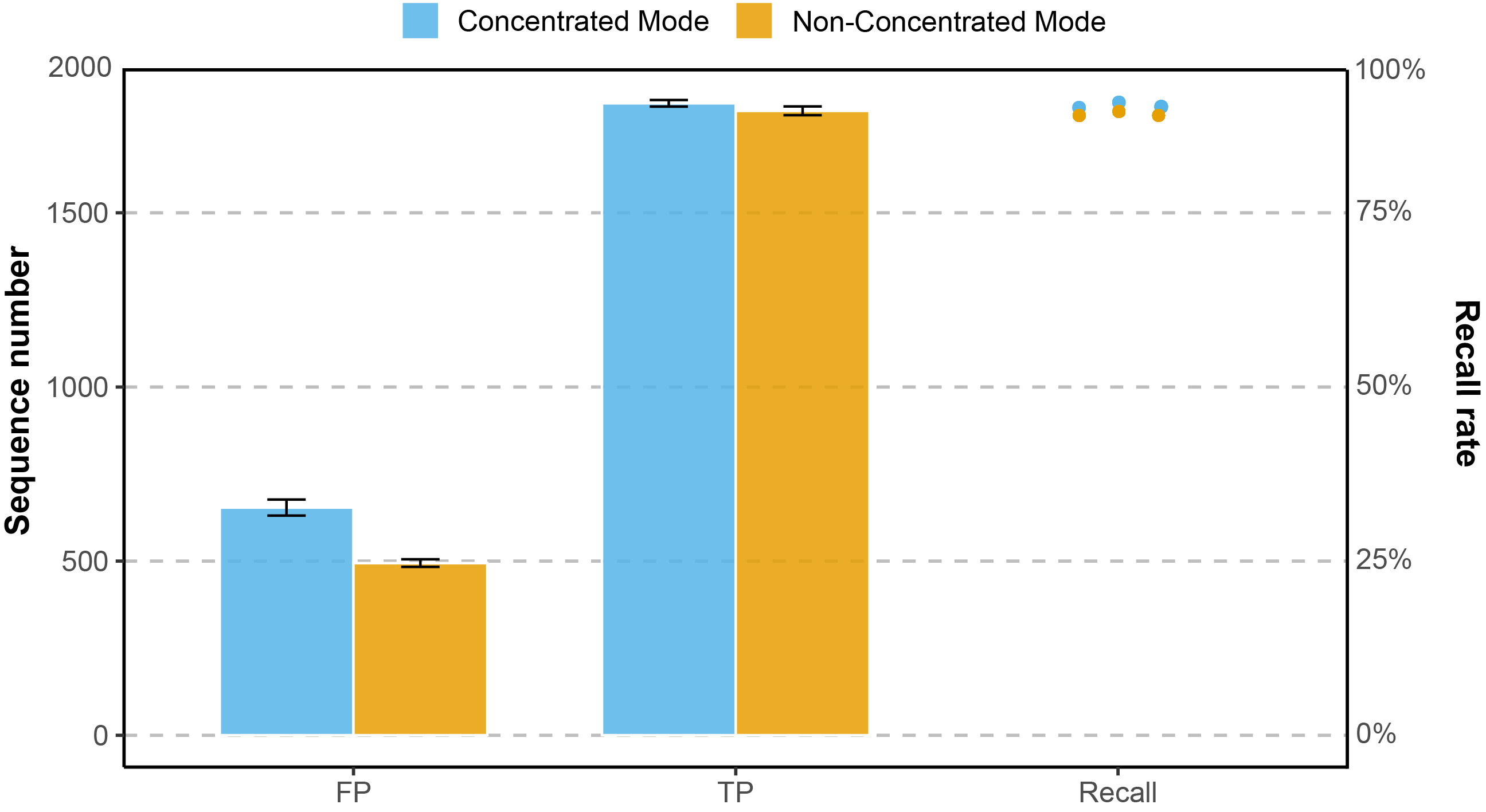
**

**Figure S4 Performance metric of ViOTUcluster “Concentrate” mode and “Non-concentrated” mode for mock samples with low-virus abundance (Low: 2000 viral sequences + 20000 non-viral sequences).** Error bars indicate the standard deviation across replicates.
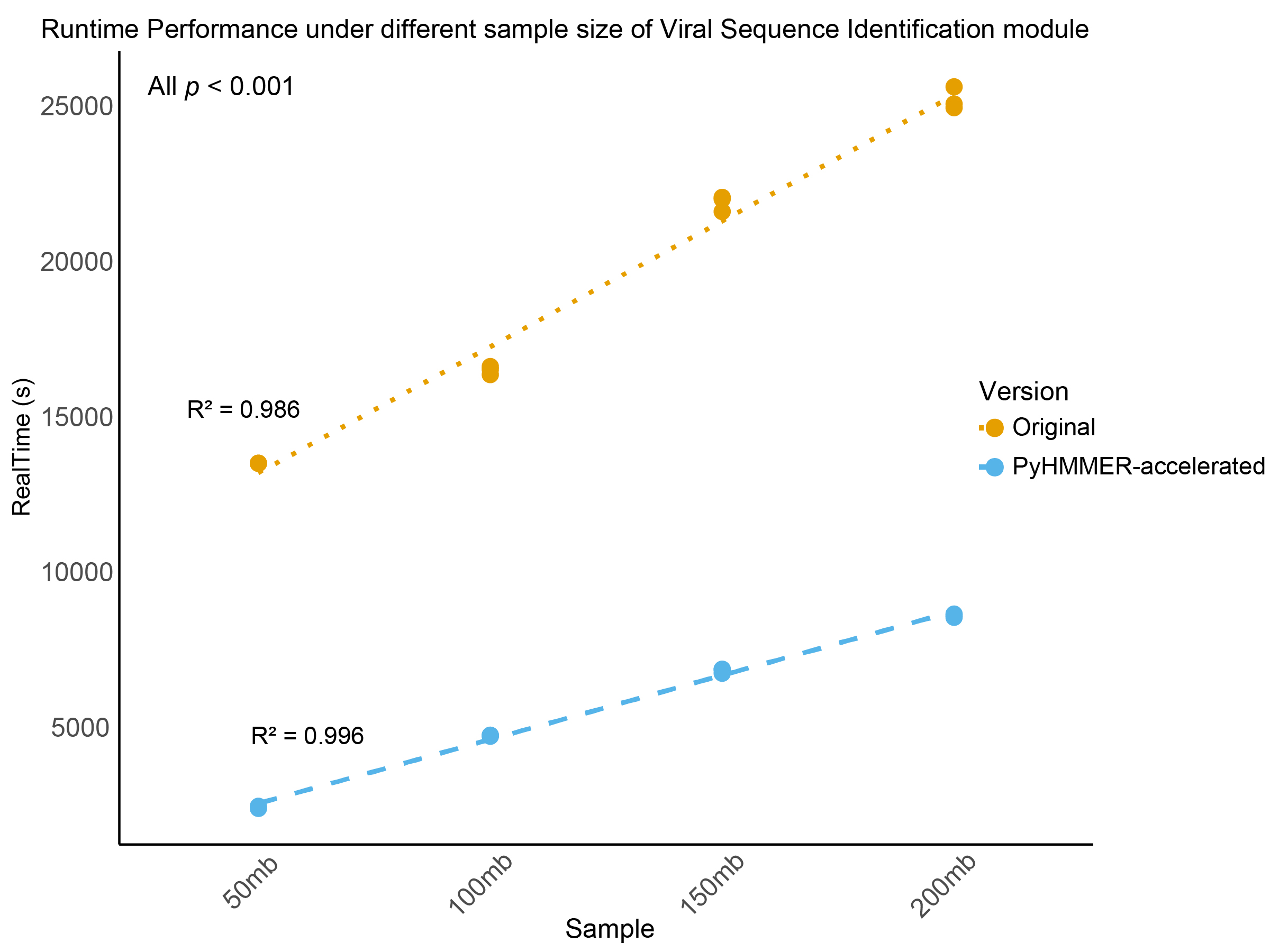


**Figure S5 Runtime performance comparison of the viral sequence identification module using the original and refactored versions of VirSorter2 and viralVerify under mock samples of varying sizes.** The curves were fitted using linear regression to model the relationship between sample size and runtime.


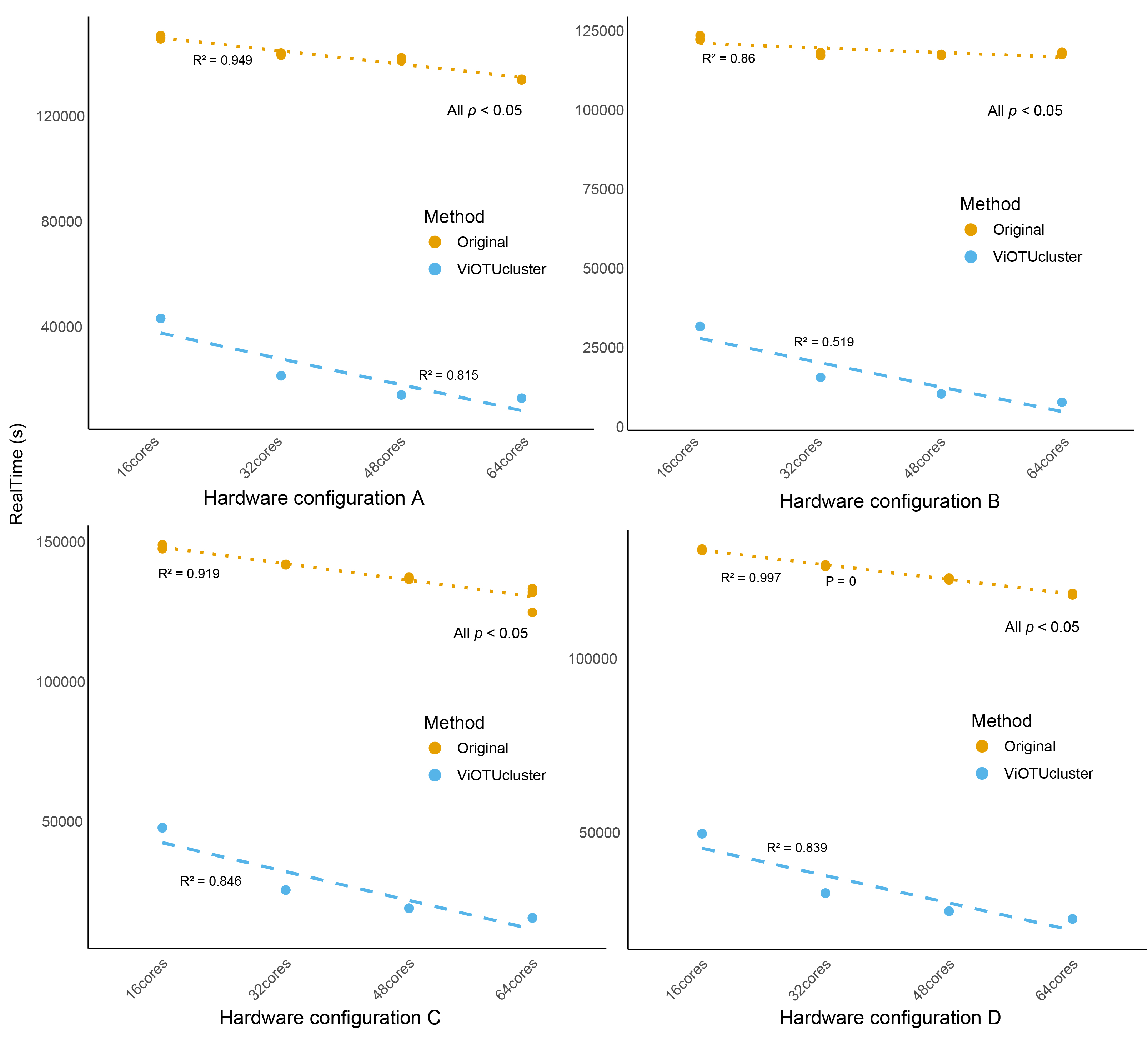


**Figure S6 Total runtime comparison between the ViOTUcluster viral sequence prediction module and the original method across different numbers of CPU cores (16 to 64) under four hardware configurations.** (A) Intel Xeon Platinum 8369B, (B) AMD EPYC Genoa 9T24; (C) Intel Xeon Platinum 8575C, and (D) AMD EPYC Milan 7T83. Detailed configuration information is provided in Table S1. The curves were fitted using linear regression to model the relationship between sample size and runtime.


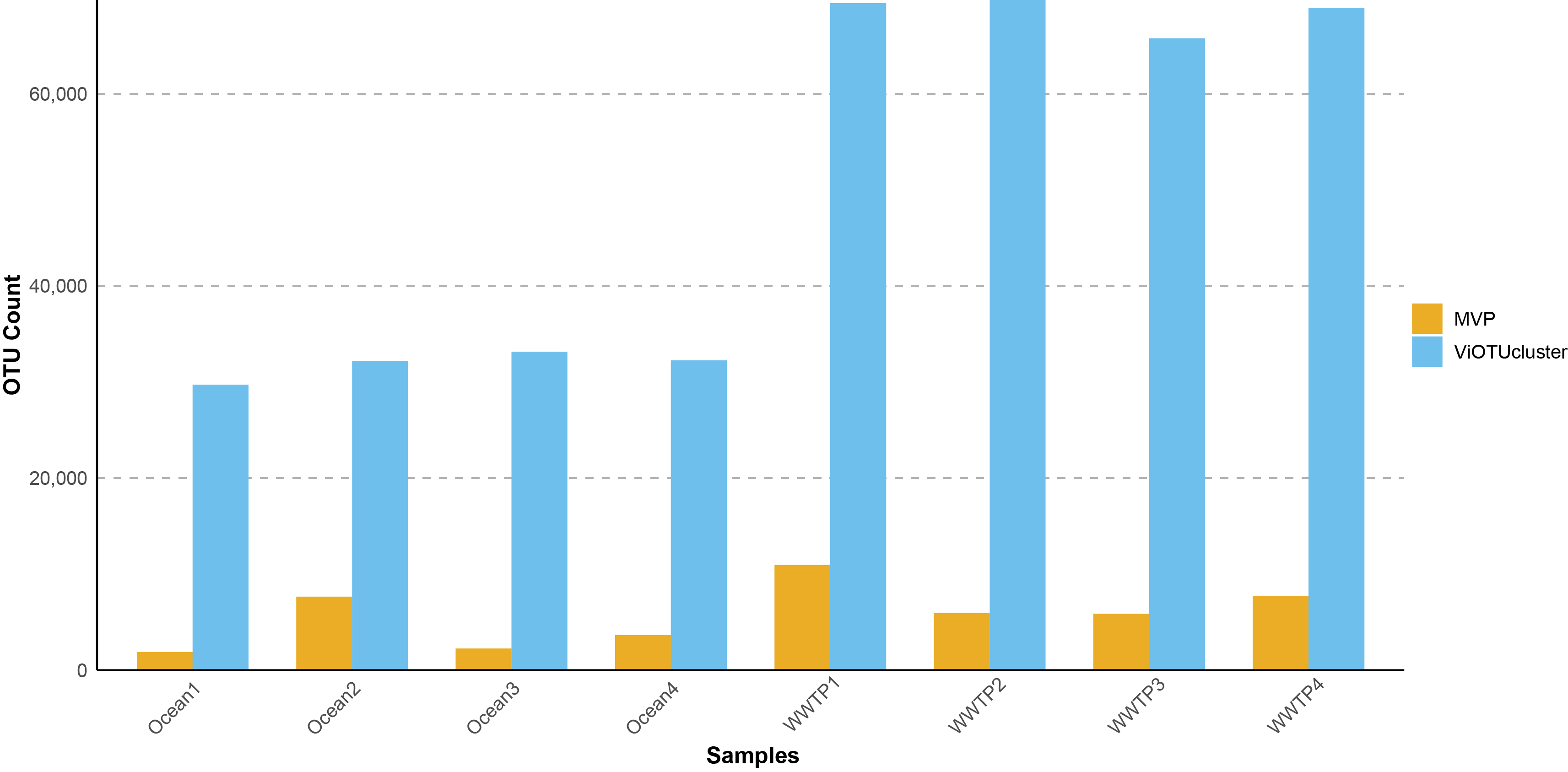


**Figure S7 The number of identified vOTUs in each sample between MVP and ViOTUcluster.** ViOTUcluster consistently recovered a greater number of vOTUs across all ocean and WWTP samples


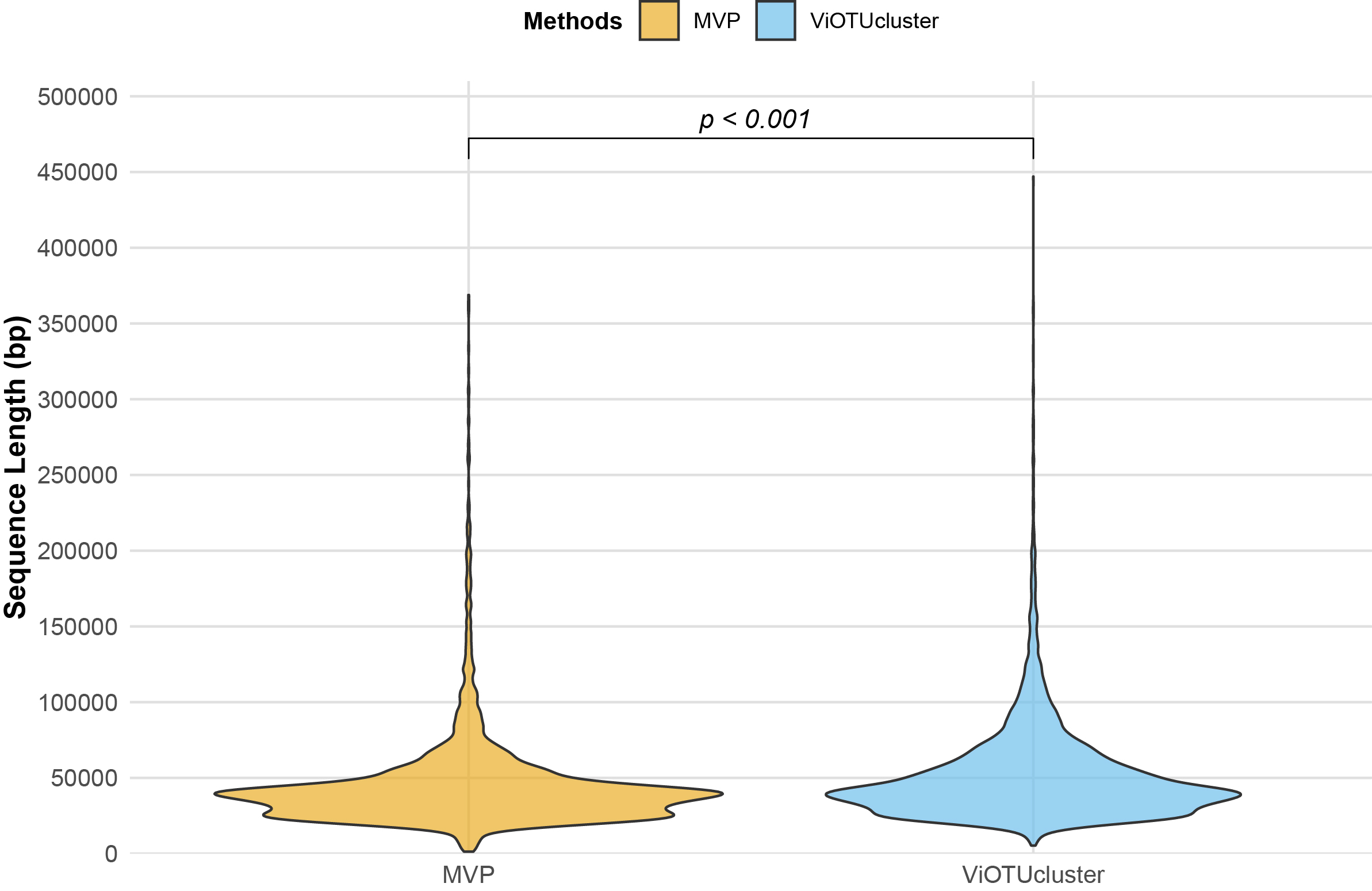


**Figure S8 Violin plot comparing medium-, high-, and complete vOTU sequence lengths (in base pairs) obtained using MVP and ViOTUcluster in ocean and WWTP samples.** The significance level was determined using the Mann-Whitney U Test.


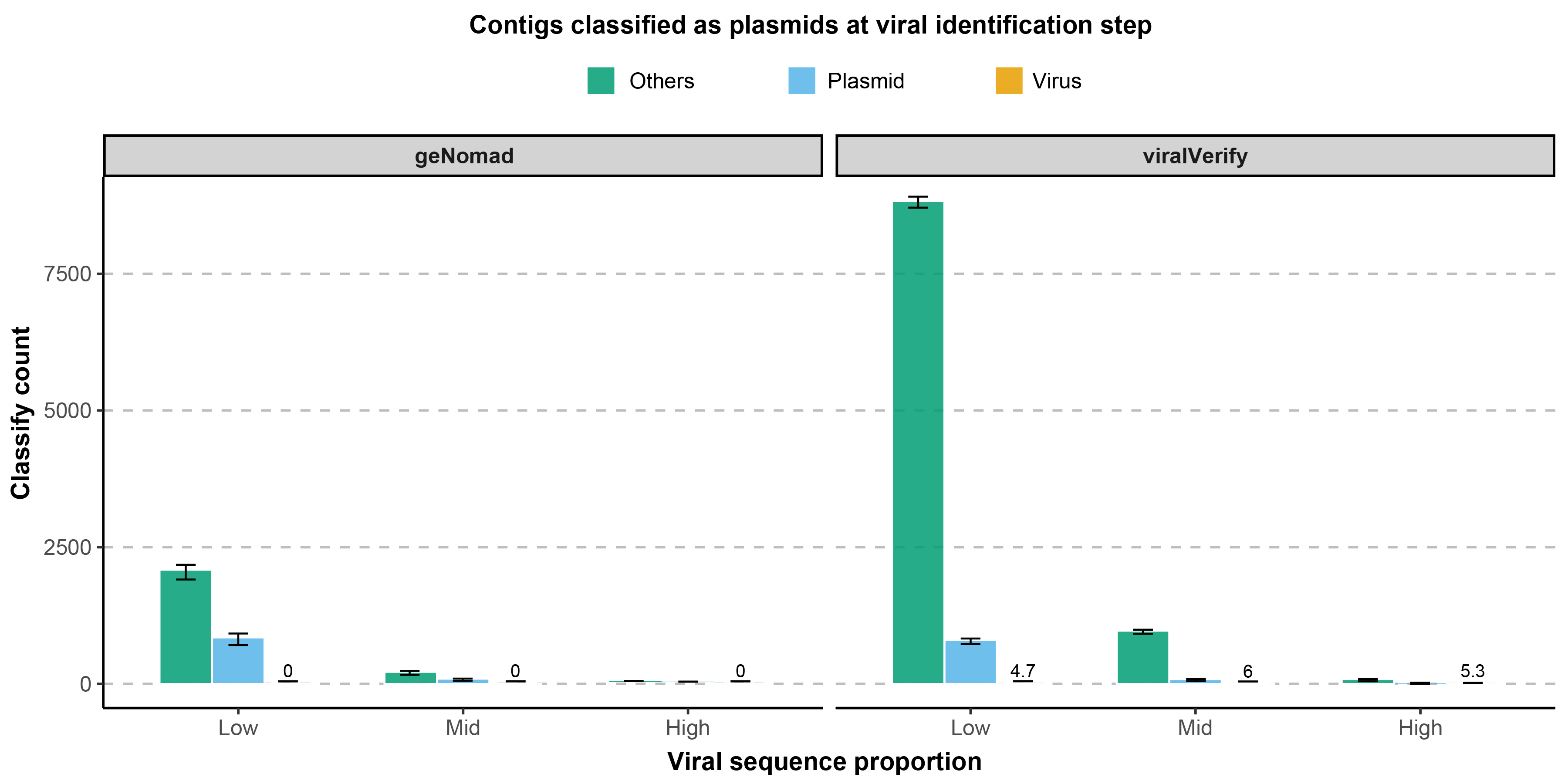


**Figure S9 The number of contigs classified as plasmids by geNomad and viralVerify in mock samples with different viral ratios (Low: 2000 viral sequences + 20000 non-viral sequences, Mid: 2000 viral sequences + 2000 non-viral sequences, High: 2000 viral sequences + 200 non-viral sequences).** Error bars represent the standard deviation of three replicates. Contigs labeled as plasmids by geNomad and viralVerify are classified into three groups: 'Others' represents non-plasmid and non-viral contigs, 'Plasmid' represents plasmid contigs, and 'Virus' represents viral contigs. The mean number of retrieved virus sequences is noted above the corresponding column.


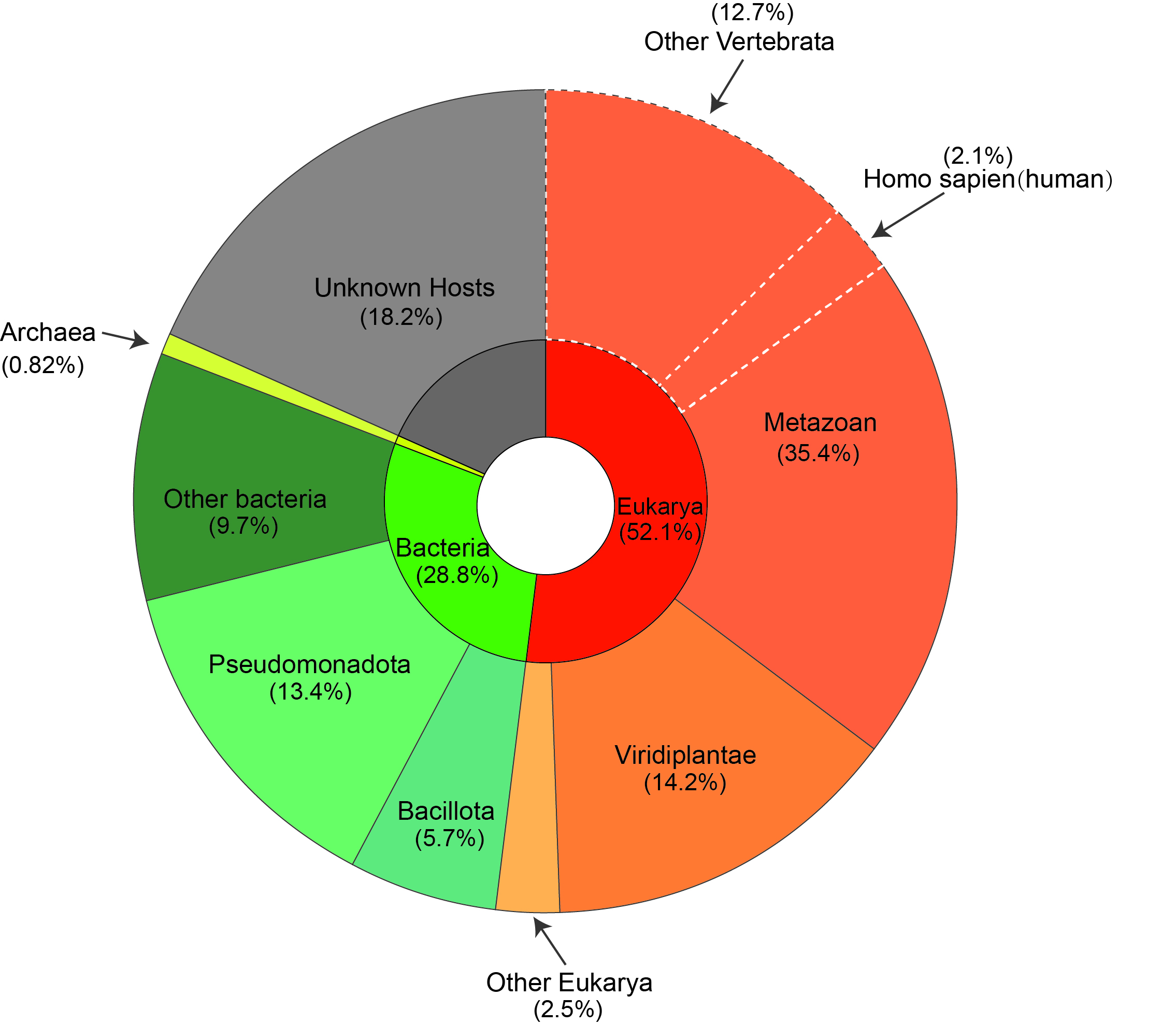


**Figure S10 Host distribution of NCBI virus Refseq database.** Each segment represents the relative proportion of viruses hosted by each group, with percentages indicated for each taxon. The inner circle represents the distribution of viral hosts at the domain level, while the outer circle shows the distribution at the kingdom level. The white borders highlight the proportion of Homo sapiens and Other Vertebrates within the total Virus RefSeq database hosts. Data accessed in March 2025.
